# The HDAC inhibitor GCJ-490A suppresses c-Met expression through IKKα and overcomes gefitinib resistance in non-small cell lung cancer

**DOI:** 10.20892/j.issn.2095-3941.2021.0130

**Published:** 2022-02-22

**Authors:** Ting He, Yinglei Gao, Yanfen Fang, Yangming Zhang, Shuwei Zhang, Fajun Nan, Jian Ding, Yi Chen

**Affiliations:** 1 Division of Anti-Tumor Pharmacology, Shanghai Institute of Materia Medica, Chinese Academy of Sciences, Shanghai 201203, China; 2 University of Chinese Academy of Sciences, Beijing 100049, China; 3 State Key Laboratory of Drug Research, the National Center for Drug Screening, Shanghai Institute of Materia Medica, Chinese Academy of Sciences, Shanghai 201203, China

**Keywords:** HDAC inhibitor, c-Met, IKKα, NSCLC, gefitinib

## Abstract

**Objective:**

The novel compound GCJ-490A has been discovered as a pan-histone deacetylase (HDAC) inhibitor that exerts potent inhibitory activity against HDAC1, HDAC3, and HDAC6. Because of the important roles of HDACs in lung cancer development and the high distribution of GCJ-490A in lung tissue, we explored the anti-tumor potency of GCJ-490A against non-small cell lung cancer (NSCLC) *in vitro* and *in vivo* in this study.

**Methods:**

The *in vitro* effects of GCJ-490A alone or combined with the EGFR inhibitor gefitinib against NSCLC were measured with proliferation, apoptosis, and colony formation assays. NSCLC xenograft models were used to investigate the efficacy of GCJ-490A combined with gefitinib for the treatment of NSCLC *in vivo*. Western blot assays, luciferase reporter assays, chromatin immunoprecipitation assays, quantitative real time-PCR, immunohistochemistry, and transcription factor activity assays were used to elucidate possible mechanisms.

**Results:**

GCJ-490A effectively inhibited NSCLC cell proliferation and induced apoptosis *in vitro* and *in vivo*. Interestingly, inhibition of HDAC1 and HDAC6 by GCJ-490A increased histone acetylation at the IKKα promoter and enhanced IKKα transcription, thus decreasing c-Met. Moreover, this c-Met downregulation was found to be essential for the synergistic anti-tumor activity of GCJ-490A and gefitinib.

**Conclusions:**

These findings highlight the promising potential of HDAC inhibitors in NSCLC treatment and provide a rational basis for the application of HDAC inhibitors in combination with EGFR inhibitors in clinical trials.

## Introduction

Non-small cell lung cancer (NSCLC) remains the most common malignancy worldwide and is a leading cause of cancer-related death^1^. Extensive efforts in recent decades have attempted to combat lung cancer, particularly through the development of epidermal growth factor receptor (EGFR) inhibitors—a milestone in NSCLC targeted therapy. EGFR inhibitors, such as gefitinib and erlotinib, provide substantial clinical benefits in patients with EGFR activation mutations (exon 19 deletion or exon 21-point mutation). However, patients eventually develop acquired resistance, which leads to rapid disease progression, thereby limiting the long-term efficacy of these agents^2^. Therefore, a need remains to continue exploring novel therapies for lung cancer.

Beyond the important roles played by kinases in cancer, epigenetic dysregulation has a key role in controlling tumorigenesis and progression^3^. As effectors of epigenetic modifications, histone deacetylases (HDACs) are associated with histone or non-histone protein deacetylation, and are functionally associated with human cancer development and progression. Consequently, several HDAC-targeting agents have been approved for cancer treatment: to date, 4 HDAC inhibitors are U.S. Food & Drug Administration (FDA) approved: SAHA, romidepsin, belinostat, and panobinostat^4^. Current research focuses on the potential utility of HDAC inhibitors as monotherapy or in combination with other chemotherapeutic regimens in NSCLC, with the aim of improving their efficacy or decreasing tumor resistance. Trichostatin A (TSA) and SAHA have been found to display strong antitumor activities in 50% of NSCLC cell lines, thus suggesting that they may have potential value in the treatment of NSCLC^5^. Existing evidence indicates that HDAC inhibitors repress lung cancer cells proliferation and overcome resistance to EGFR inhibitors. HDAC6 is over-expressed in lung adenocarcinoma cell lines, and it promotes NSCLC proliferation and resistance to gefitinib^6^. Inhibition of HDAC6 deacetylase activity by CAY10603, a selective HDAC6 inhibitor, sensitizes NSCLC to gefitinib^6^. YF454A, a new compound targeting HDACs, has also been reported to inhibit the growth of EGFR-TKI-resistant NSCLC in synergy with erlotinib by blocking cell cycle pathways and the receptor tyrosine kinase pathway^7^.

In working with chemists to develop novel HDAC inhibitors, we discovered the novel pan-HDAC inhibitor GCJ-490A (referred to as H13 in a previous publication)^8^. The compound has strong inhibitory effects on HDAC1, HDAC3, and HDAC6. At the molecular level, the half-maximal growth inhibitory concentration (IC_50_) of GCJ-490A is as low as the nanomolar range, a level comparable to that of the HDAC inhibitor LBH589^9,10^. In addition, this agent has a favorable PK profile and high tissue distribution specificity in the digestive tract, colon, and lung^8^. Because of the high distribution of this compound in the lung and the potential utility of HDAC inhibitors in treating lung cancer, we explored the potential of GCJ-490A in the treatment of NSCLC in the present study.

## Materials and methods

### Compounds

Gefitinib (ZD1839) was purchased from Selleck Chemicals (Houston, TX, USA). Compounds were dissolved in DMSO at a concentration of 10 mM and stored at −80 °C for *in vitro* assays.

### Cell culture

The NSCLC cell lines A549, H460, H23, H1975, PC9, H1792, H1993, H1299, H2228, and HCC366, as well as HEK293T embryonic kidney cells, were purchased from ATCC (Manassas, VA, USA). HCC827 and HCC827/GR6 were kindly provided by Dr. Pasi A. Jänne (Dana-Farber Cancer Institute, Boston, MA, USA). All cells were maintained according to the providers’ instructions.

### Proliferation assays

Cell number was monitored with Sulforhodamine B (Sigma-Aldrich #S9012, St. Louis, MO, USA) according to the manufacturer’s instructions. The doses corresponding to the IC_50_ values were calculated with SoftMax software.

### Determination of synergism

Synergistic effects of 2 drugs, as determined by the combination index (CI) values, were calculated with Calcusyn software. CI values of < 0.8, 0.8, and > 0.8 indicated synergistic, additive, and antagonistic effects, respectively^11^. We also calculated CI values with the IC_50_ ratio obtained with GCJ-490A plus gefitinib compared with that obtained with gefitinib alone, as previously described^12^.

### Colony formation assays

Cells in logarithmic growth phase were digested with 0.25% trypsin solution and separated into single cells. Cells (1 × 10^3^ cells/4 mL/well) were seeded in 6-well plates, cultured overnight, and treated with the indicated agents for 12 days. The culture medium was changed twice during the period, and the final concentration of the drug in each well was consistently maintained. Then the cells were fixed with solution containing 10% methanol and 10% acetic acid for 30 min at room temperature. Next, the cells were stained with 1 mL of 1% crystal violet at room temperature, dried, scanned, and counted. These experiments were performed in triplicate, and statistical significance was assessed with Student’s *t*-test.

### Apoptosis assays

The percentage of apoptotic cells was determined with an Annexin V-FITC/PI double staining kit (C1052) (Vazyme Biotech, Nanjing, China) and analyzed in BD FlowJo software. Each experiment was performed in triplicate.

### Western blot

Cells were processed in RIPA buffer containing Complete mini protease inhibitor cocktail (11836153001) (Roche, Basel, Switzerland) and phosphatase inhibitors (B15002) (Bimake, Houston, TX, USA). Cell extracts were probed with primary antibodies to the following: histone 3 (17168-1-AP) (Proteintech, IL, USA); histone H3 (acetyl K9) (ab10812) (Abcam, Cambridge, UK); acetylated α-tubulin (sc-23950) (Santa Cruz Biotechnology, CA, USA); α-tubulin (3873S), β-actin (4970S), GAPDH (5174S), PARP (9542S), cleaved PARP (5625S), Bcl-XL (2764S), caspase-3 (9665S), cleaved caspase-3 (9664S), Bim (2933S), c-Met (8198S), phospho-c-Met (Tyr1234/1235) (3077S), phospho-NF-κB RelA/p65 (Ser536) (3033S), NF-κB RelA/p65 (8242S), TBP (44059S), IKKα (11930S), HDAC1 (34589S), HDAC3 (85057S), and HDAC6 (7558S) (Cell Signaling Technology, MA, USA). Subsequently, the bound antibodies were detected with enhanced chemiluminescence detection reagent (Thermo Fisher Scientific, IL, USA) with a ChemiDoc MP System (Bio-Rad Laboratories, CA, USA).

### Immunohistochemistry (IHC) and TUNEL staining

The tumor tissue was fixed in 4% paraformaldehyde. All specimens were embedded in paraffin and sliced into 10 µm thick sections. After dewaxing and hydration, the sections were incubated in 3% H_2_O_2_ to eliminate endogenous peroxidase activity. Then the sections were blocked with 0.5% bovine serum albumin for 1 h at room temperature. The sections were subsequently incubated overnight at 4 °C with anti-Ki-67 (8D5) (Cell Signaling Technology #9449S), anti-histone H3 (acetyl K9) (Abcam #ab10812), anti-c-Met (Cell Signaling Technology #8198S), anti-IKKα (Abcam #ab32041), and anti-Bcl-XL (Cell Signaling Technology #2764S). The sections were observed under a light microscope. TUNEL staining was performed with an *in situ* cell death detection kit, POD (Roche #11684817910) according to the manufacturer’s protocol. The same quantification method used for the IHC analyses was applied in the TUNEL analysis. The relative fluorescence intensity and the number of IHC-positive cells in each section were measured and quantified in NDP.view software.

### RNA interference

RNA oligonucleotides (GenePharma, Shanghai, China) (**Table 1**) and RNAiMAX Transfection Reagent (13778-150) (Invitrogen, CA, USA) were used for RNA interference (RNAi) assays.

**Table 1:** The siRNA target sequences

RNA oligonucleotides	Sense (5′–3′)
*HDAC1*-Homo-1	GCUGUACAUUGACAUUGAUTT
*HDAC1*-Homo-2	CCGGUCAUGUCCAAAGUAATT
*HDAC1*-Homo-3	GCUCCUCUGACAAACGAAUTT
*HDAC3*-Homo-1	CCAAGAGUCUUAAUGCCUUTT
*HDAC3*-Homo-2	GCAUCUCUGCAAGGAGCAATT
*HDAC3*-Homo-3	GCCGCUACUACUGUCUGAATT
*HDAC6*-Homo-1	GCAAUGGAAGAAGACCUAATT
*HDAC6*-Homo-2	CCGUGGAGAGGGACAACAUTT
*HDAC6*-Homo-3	GAGGACAAUGUAGAGGAGATT
*p65*-Homo-1	CAGAUACAGACGAUCGUCATT
*p65*-Homo-2	CCCUAUCCCUUUACGUCAUTT
*p65*-Homo-3	GAUGAAGACUUCUCCUCCATT
*IKK*α-Homo-1	GACCAUGGUAUUUGAAUGUTT
*IKK*α-Homo-2	CCUGGCAUGAGAAGAUUAATT
*IKK*α-Homo-3	GCUGUAAGCAGAAGAUUAUTT
*c-Met*-Homo-1	GGACAATGATGGCAAGAAA
*c-Met*-Homo-2	CAAAGAAGGAAGTGTTTAA
*c-Met*-Homo-3	CCACGTGAACGCTACTTAT

### Lentivirus production and *in vitro* infection

Lentiviruses were produced by co-transfection of 293T cells with plasmid DNA (**Table 2**) and packaging vectors (pmd2.g and pax2) with Lipofectamine 2000 (Invitrogen #11668-019). Supernatant was collected 48 and 72 h post-transfection, concentrated by ultracentrifugation at 30,000 rpm for 120 min, and resuspended in an appropriate volume of OptiMEM (Gibco #22600134). After being counted, the target cells were seeded into 6-well plates (2 × 10^5^ cells in 500 µL/well) and infected with an appropriate number of lentiviruses for 48 h.

**Table 2:** Plasmid information

Lentiviral plasmid	Element
lenti-*p65*	pLenti-EF1a-EGFP-P2A-Puro-CMV-p65-3Flag
*p65*_MOCK	pLenti-EF1a-EGFP-P2A-Puro-CMV-MCS-3Flag
sh*p65*	pLKD-CMV-EGFP-2A-Puro-U6-shRNA (p65)
sh*p65*_MOCK	pLKD-CMV-EGFP-2A-Puro-U6-shRNA
lenti-*IKKa*	pRLenti-EF1a-EGFP-P2A-Puro-CMV-IKKα-3Flag
*IKKa*_MΟCK	pRLenti-EF1a-EGFP-P2A-Puro-CMV-MCS-3Flag
sh*IKKa*	pLKD-CMV-EGFP-2A-Puro-U6-shRNA (IKKα)
sh*IKKa*_MOCK	pLKD-CMV-EGFP-2A-Puro-U6-shRNA

### Quantitative real time-PCR (RT-PCR)

Total RNA was extracted from cells with TRIzol reagent (Thermo Fisher Scientific #15596-018). The cDNA was prepared with a HiScript II Q RT SuperMix for qPCR kit (Vazyme #R223-01) according to the manufacturer’s protocol. Quantitative RT-PCR gene expression analyses were performed in duplicate with iTaq Universal SYBR Green Supermix (Bio-Rad #1725124) by using a Real-Time PCR System (VIIA7) (ABI, MA, USA). Gene expression data were normalized to *GAPDH* mRNA expression, and the RQ values were used to analyze the gene expression among groups. Independent experiments were repeated at least 3 times. Information on the primer sequences is provided in **Table 3**.

**Table 3:** Primer sequences

Gene name	5′–3′
*c-Met*: F	ACCTTTGATATAACTGTTTACTTGTTGCA
*c-Met*: R	GCTTTAGGGTGCCAGCATTTT
*RELA/p65*: F	ATGTGGAGATCATTGAGCAGC
*RELA*/*p65*: R	CCTGGTCCTGTGTAGCCATT
*IKK*α: F	ATGAAGAAGTTGAACCATGCCA
*IKK*α: R	CCTCCAGAACAGTATTCCATTGC
*HDAC1*: F	CTACTACGACGGGGATGTTGG
*HDAC1*: R	GAGTCATGCGGATTCGGTGAG
*HDAC3:* F	CCTGGCATTGACCCATAGCC
*HDAC3:* R	CTCTTGGTGAAGCCTTGCATA
*HDAC6:* F	CGGGAAGTCGCGGGGAAAA
*HDAC6:* R	CGCTTCGAAGTGACACTGGAG
*GAPDH*: F	CCACCCATGGCAAATTCCATGGCA
*GAPDH*: R	TCTAGACGGCAGGTCAGGTCCACC

### NF-κB RelA/p65 transcription factor binding assays

An NF-κB p65 (human) transcription factor activity assay kit (E-4330-100) (BioVision, CA, USA) was used to quantify active NF-κB p65 *in vitro*. According to the manufacturer’s instructions, cell samples treated with the indicated compounds were added to double-stranded oligonucleotides (containing the NF-κB binding sequence) in coated 96-well plates. Antibodies to NF-κB p65 and HRP-conjugated secondary antibodies were separately added to each well. Finally, the plate was read at 450 nm.

### Nuclear and cytoplasmic protein separation

Nuclear and cytoplasmic proteins were extracted with a nuclear and cytoplasmic protein extraction kit (P0028) (Beyotime Biotechnology, Shanghai, China) according to the manufacturer’s protocol. Proteins were analyzed by Western blot.

### Chromatin immunoprecipitation (ChIP) assays

The ChIP assays were performed with a Simple ChIP Plus Enzymatic Chromatin IP kit (Cell Signaling Technology #9005) according to the manufacturer’s recommendations. Immunoprecipitated DNA was used as a template for real time quantitative PCR reactions. Antibodies and oligonucleotides used as primers (**Tables 4 and 5**) in ChIP assays were as follows: NF-κB p65 (Cell Signaling Technology #8242S) and anti-histone H3 (acetyl K9) (Abcam #ab10812).

**Table 4:** Primer sequences in the *c-Met* promoter region

*c-Met* promoter region	Sense (5′–3′)	Anti-sense (5′–3′)
−1819 to −1547	TATGGAAACCAGGAAATAGAAACAG	CTGACTGGAGATTTCCTGATACGGC
−1505 to −1320	TGTGAAGGACACCTGACTGGGCTGA	ATTACCTCTTGATTCCCCAGTTTTA
−1111 to −855	GATTGAACAAGTTGGTATGAGAGCC	AAATAGCGATGAATAAGCACAACAG
−868 to −620	ATTCATCGCTATTTGCCCAGTTATT	GTTTAGAGAGATTTGGGCACCGCAG

**Table 5:** Primer sequences in the *IKKa* promoter region

*IKK*α promoter Region	Sense (5′–3′)	Anti-sense (5′–3′)
−1206 to −892	TGTTTCCCCTATGCTATGGCAATCC	GGGTTTGCCAGCACAGTAGTTCATT
−930 to −783	GAAACACTTCTCACAATGAACTACT	AAATGACTCAAGAATGTGACGCTAT
−805 to −738	AGCGTCACATTCTTGAGTCATTTGG	ACTGACTTCCCAATACACCTGCTCC
−761 to −616	GAGCAGGTGTATTGGGAAGTCAGTG	ATCTATCCTCCAACTCCACCACCAG
−640 to −481	CTGGTGGTGGAGTTGGAGGATAGAT	AAGTAGTCCTCCCACCTTGGCATCC

### Luciferase reporter assays

HEK293T embryonic kidney cells were transfected with *c-Met* promoter, transcription factor RelA/p65, or control plasmids alone or together, by using Lipofectamine 2000 (Invitrogen #11668-019). Six hours after transfection, cells were cultured in fresh culture medium for 42 h. *Renilla* and firefly luciferase activity were analyzed with the Dual-Luciferase Reporter Assay System (E1910) (Promega, WI, USA). Data were normalized to *Renilla* luciferase activity.

### RNA-sequencing analysis

A549 and HCC827/GR6 non-small lung cancer cells were treated with GCJ-490A at a final concentration of 50 nM for 2 days. Total RNA was extracted from cells with TRIzol reagent (Thermo Fisher Scientific #15596-018). The transcriptome analysis of 12 samples was performed with next-generation sequencing. The clean data for each sample exceeded 7.91 GB, and the percentage of the q30 base exceeded 94.37%. Next, we compared the clean reads of each sample with the *Homo sapiens* genome. The comparison rate ranged from 97.25% to 98.0%. On the basis of the above qualified sample information, differentially expressed genes between 2 groups were analyzed in DESeq2 software. *P*-values obtained from our tests were adjusted with the Benjamini-Hochberg correction. Significantly differentially expressed genes were defined by a Benjamini-Hochberg corrected *P-*value cutoff of 0.01 and a fold-change of at least 1. To obtain the bubble chart, we performed GO enrichment analysis of differential genes with Goatools. Gene set enrichment analysis (GSEA) was performed to rank the degree of differential expression between the control group and treatment groups.

### *In vivo* studies

All experiments were performed according to the institutional ethical guidelines on animal care and were approved by the Institute of Animal Care and Use Committee at Shanghai Institute of *Materia Medica* (Approval Nos. 2017-04-DJ-26 and 2018-05-DJ-37). Nude mice were subcutaneously injected with human NSCLC cells (5 × 10^6^ cells/mouse) into the right flank, and tumor bearing mice were then randomized into groups. Treatments started when the tumor reached an average volume 100–200 mm^3^. Mice were treated with vehicle, GCJ-490A (0.5% CMC-Na), gefitinib (0.9% NaCl), or both by daily oral gavage. Tumor diameters were measured twice per week, and the relative tumor volumes (RTV) were calculated with the formula: RTV = (½ × length × width^2^ of day n)/(½ × length × width^2^ of day 0). The therapeutic effect was evaluated according to the RTV ratio of treatment to vehicle control: T/C (%) = 100% × (mean RTVtreated)/(mean RTVvehicle).

### Statistical analysis

Two-tailed Student’s *t*-test was used for comparisons between 2 groups, and one-way ANOVA was applied for multiple group statistical comparisons in GraphPad Prism 6 (GraphPad Software). Unless otherwise indicated, the results are expressed as the mean ± SD from at least 3 independent experiments. Values of *P* < 0.05 were considered statistically significant: *P < 0.05; **P < 0.01; ***P < 0.001; ^#^*P* < 0.05; ^##^*P* < 0.01; ^###^*P* < 0.001; ^&^*P* < 0.05; ^&&^*P* < 0.01; ^&&&^*P* < 0.001 (*P* values are indicated by */#/&).

## Results

### GCJ-490A inhibits NSCLC cell proliferation *in vitro* and *in vivo*

**Figure 1:**
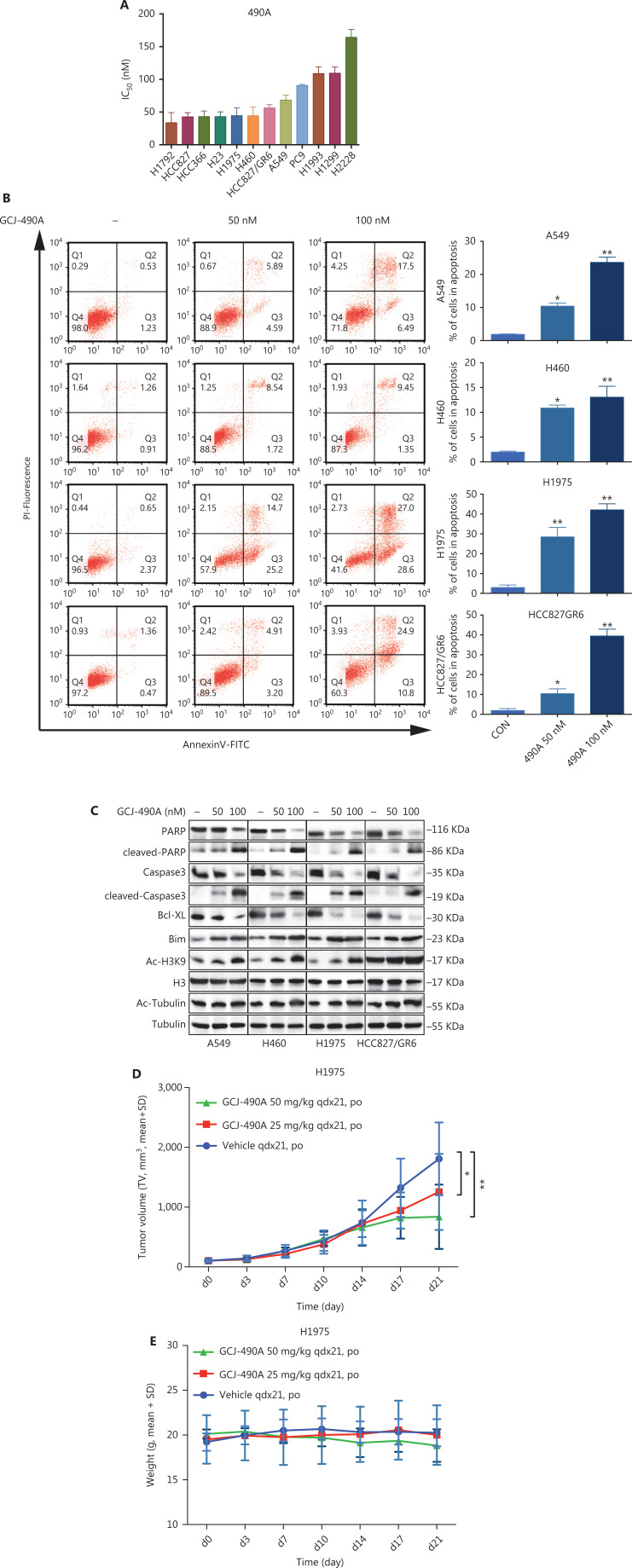
GCJ-490A inhibits NSCLC cell proliferation *in vitro and in vivo.* (A) IC_50_ values for exposure of NSCLC cell lines to GCJ-490A for 72 h. (B) Assessment of apoptosis by FACS analysis. NSCLC cells were treated with GCJ-490A for 48 h. The sum of annexin V^+^/PI^-^ and annexin V^+^/PI^+^ labeled cells was counted as the total apoptotic cells. (C) The levels of apoptosis-related proteins were detected in A549, H460, H1975, and HCC827/GR6 cells treated with GCJ-490A for 48 h. (D) Antitumor activity of GCJ-490A *in vivo*. Tumor growth curve graphs show relative tumor volume (RTV) over time in each treatment group. (E) Body weights of animals in each treatment group over time. All *in vitro* data are presented as the mean ± SD from at least 3 independent experiments, and were analyzed by 2-tailed Student’s *t*-test, *P < 0.05; **P < 0.01; 490A: GCJ-490A, GEFI: gefitinib.

A panel of NSCLC cell lines was used to assess the activity of the HDAC inhibitor GCJ-490A against human NSCLC. As expected, GCJ-490A markedly inhibited the proliferation of all tested NSCLC cell lines in a dose-dependent manner, including cells reported to be gefitinib resistant, such as A549, H460, HCC827/GR6, and H1975, with IC_50_ values ranging from 20 to 180 nM/L (**Figure 1A**). HDAC inhibitors have been reported to induce apoptosis in NSCLC^13^; therefore, we evaluated the effect of GCJ-490A on apoptosis. This agent led to apoptosis in a dose-dependent manner, as indicated by flow cytometric analysis (Annexin V^+^/PI^-^ and Annexin V^+^/PI^+^ cells) (**Figure 1B**). GCJ-490A also induced an increase in cleaved-PARP, activated caspase3, and the pro-apoptotic protein Bim, and a decrease in the anti-apoptotic protein Bcl-XL, which was accompanied by up-regulation of acetylated histone H3 lysine 9 (H3K9-Ac) and acetylated α-tubulin (α-tubulin-Ac). We observed substantial cellular HDAC inhibition in 4 tested NSCLC cell lines: A549, H460, H1975, and HCC827/GR6 (**Figure 1C**). We attempted to detect whether GCJ-490A might suppress NSCLC growth *in vivo*. As shown in **Figure 1D**, GCJ-490A inhibited tumor growth in an NSCLC xenograft model, NCI-H1975. GCJ-490A, compared with vehicle treatment, inhibited tumor growth in a dose-dependent manner, yielding a T/C rate of 48.13% at 50 mg/kg (*vs.* vehicle, *P* < 0.01) and 69.01% at 25 mg/kg (*vs.* vehicle, *P* < 0.05). None of the mice showed any macroscopic adverse effects (**Figure 1E**).

### GCJ-490A suppresses c-Met expression, thus overcoming gefitinib resistance *in vitro* and *in vivo*

**Figure 2:**
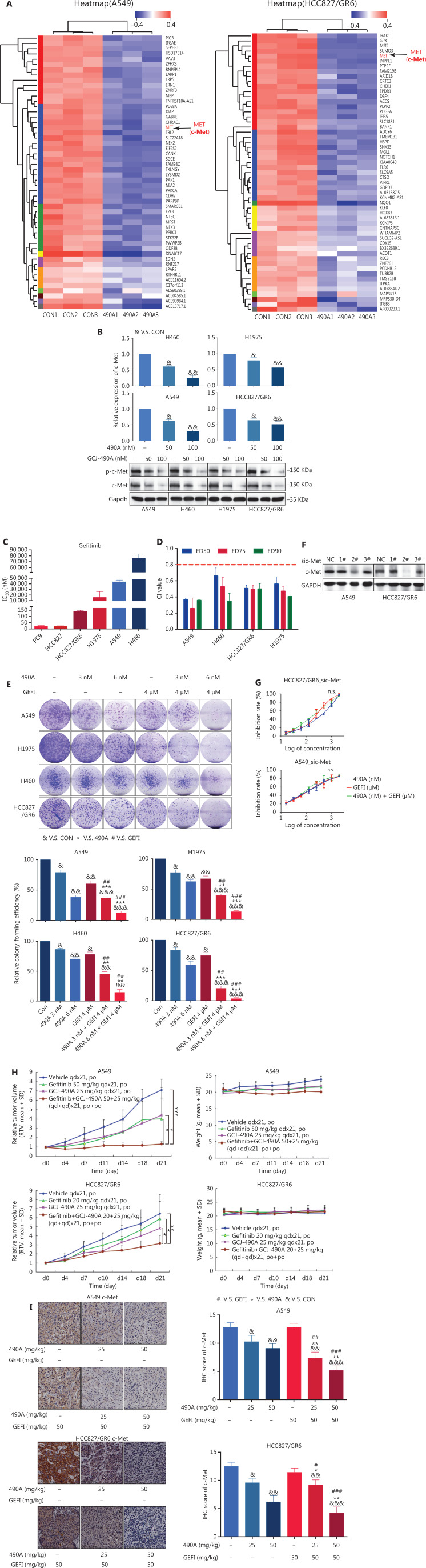
GCJ-490A suppresses c-Met expression, thus overcoming gefitinib resistance *in vitro and in vivo.* (A) RNA-seq profiling of gene expression in A549 and HCC827/GR6 cells treated with GCJ-490A. (B) Validation of the RNA-seq analysis results by quantitative real time-PCR and Western blot. (C) IC_50_ values of NSCLC cell lines after exposure to gefitinib for 72 h. (D) Average CI values. NSCLC cells were treated with GCJ-490A and gefitinib alone or in combination for 72 h. (E) Colony formation assays. NSCLC cells were treated with GCJ-490A and gefitinib alone or in combination for 2 weeks. Depletion of c-Met in NSCLC cells by sic-Met (F) and 2# sic-Met-transfected cells treated with GCJ-490A, gefitinib, or both for 72 h (G). (H) Relative tumor volume (RTV) and average body weight in nude mice treated with gefitinib and GCJ-490A alone or together in 2 xenograft models. (I) The levels of c-MET in A549 and HCC827/GR6 tumor tissues at the end of the *in vivo* studies were detected by IHC assays (scale bar 100 μm). All *in vitro* experiments were repeated 3 times and yielded closely comparable results. The data are presented as the mean ± SD, *P < 0.05; **P < 0.01; ***P < 0.001, ^#^*P* < 0.05; ^##^*P* < 0.01; ^###^*P* < 0.001; ^&^*P* < 0.05; ^&&^*P* < 0.01; ^&&&^*P* < 0.001, 490A: GCJ-490A, GEFI: gefitinib, NC: negative control; #1, #2, #3 are 3 different RNAi oligonucleotides.

We found that GCJ-490A was effective against gefitinib-resistant cell lines. Therefore, we selected 2 resistant cell lines, A549 and HCC827/GR6, for RNA-seq profiling to identify the mechanism underlying the anti-tumor effect of GCJ-490A. *c-Met* was among the transcripts most significantly downregulated by GCJ-490A in HCC827/GR6 and A549 cells (**Figure 2A, Supplementary Tables S1** and **S2**). c-Met has a well-documented role as an oncogenic driver and a driver of acquired EGFR inhibitor resistance in lung cancer^14–16^. We substantiated this result by detecting the mRNA and protein levels of c-Met in NSCLC cells. As observed in the RNA-seq profiling data, *c-Met* transcription was repressed in GCJ-490A treated NSCLC cells (**Figure 2B**). Western blot assays showed that c-Met and phosphorylated c-Met were noticeably down-regulated in GCJ-490A treated cells compared with control cells (**Figure 2B**).

Because of the function of c-Met in lung cancer^16^, we explored the possibility of GCJ-490A synergy with gefitinib, and the possibility of overcoming gefitinib resistance. As expected, gefitinib dramatically inhibited cell viability in PC9 and HCC827, with IC_50_ values less than 10 nM/L (**Figure 2C**). In contrast, similarly to findings reported in the literature, HCC827/GR6, H1975, A549, and H460 cells were resistant to gefitinib, with high IC_50_ values (**Figure 2C**). The effect of the combination therapy was determined by calculating the CI: a value less than 0.8 indicates synergy^11^. The mean CI values in these gefitinib-resistant NSCLC cells (A549, H460, HCC827/GR6, and H1975) from 3 independent experiments were all less than 0.8 (**Figure 2D**), indicating a synergistic effect between GCJ-490A and gefitinib in gefitinib-resistant cells. Similar results were obtained in colony formation assays (**Figure 2E**) and growth curve assays (**Supplementary Figure S1A**). We next confirmed the role of c-Met in combination therapy. We silenced c-Met with different siRNAs in HCC827/GR6 and A549 cells. The cells transfected with 2# sic-Met were then selected and treated with GCJ-490A and gefitinib alone or in combination (**Figure 2F**). As expected, loss of c-Met reversed the synergistic inhibitory effect of GCJ-490A plus gefitinib on cell proliferation (**Figure 2G**). These results suggested that the down-regulation of c-Met was essential for the combination therapy to overcome gefitinib resistance. We next evaluated whether GCJ-490A might enhance the anti-tumor activity of gefitinib *in vivo*. As shown in **Figure 2H**, GCJ-490A alone moderately suppressed tumor growth in 2 tested NSCLC xenograft models, HCC827/GR6 and A549. Gefitinib alone had almost no effect on tumor growth, as expected in the HCC827/GR6 model, and exhibited moderate inhibitory effects against tumor growth in the A549 model. However, combinatorial therapy with GCJ-490A and gefitinib inhibited tumor growth more potently than treatment with GCJ-490A or gefitinib alone, yielding a T/C rate of 49.00% in HCC827/GR6 (*vs*. monotherapy, *P* < 0.05) and 18.81% in A549 model (*vs*. monotherapy, *P* < 0.05), respectively (**Figure 2H, Supplementary Figure S2**). No mice treated with these agents showed any macroscopic adverse effects, including loss of body weight (**Figure 2H**). We also found that GCJ-490A and co-treated xenografts, compared with those treated with vehicle, showed lower c-Met protein expression in tumor tissues, on the basis of IHC staining (**Figure 2I**), and up-regulation of acetylated histone H3 lysine 9 (H3K9-Ac) (**Supplementary Figure S1B**), thus indicating HDAC inhibition in xenografts. The effect of combination therapy on apoptosis *in vivo* was analyzed by TUNEL staining, and the score in the combination group was significantly higher (**Supplementary Figure S1C**). In agreement with TUNEL data, IHC staining indicated that levels of Ki67 and Bcl-XL potently declined in xenografts after treatment with GCJ-490A and gefitinib together (**Supplementary Figure S1D, S1E**). These data together suggest that the decrease in c-Met expression by GCJ-490A overcomes gefitinib resistance in NSCLC.

### GCJ-490A decreases c-Met expression through an IKKα/NF-κB pathway dependent mechanism

**Figure 3:**
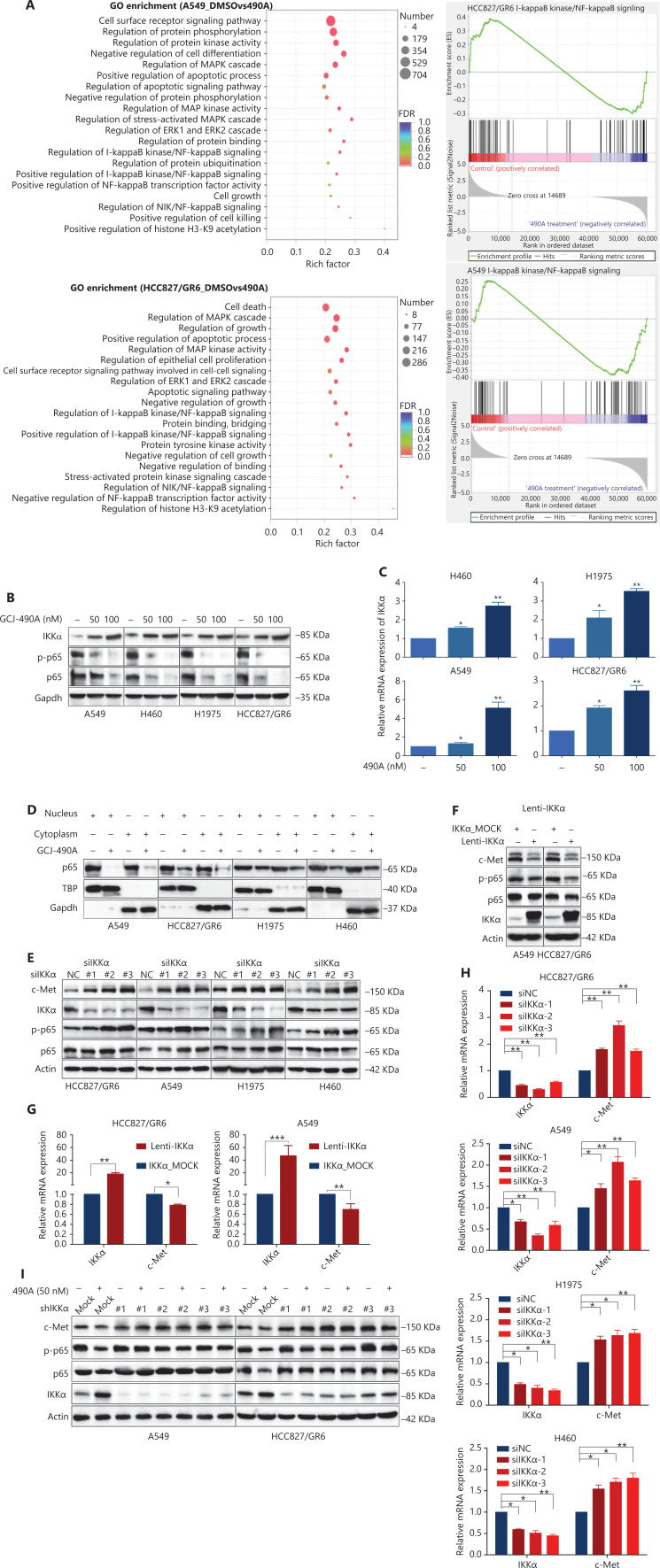
GCJ-490A decreases c-Met expression in a manner dependent on the IKKα/NF-κB pathway. (A) GO enrichment analysis and gene set enrichment analysis (GSEA). The NF-κB pathway was found to be enriched through analysis of significantly changed genes in RNA-seq profiling. GSEA plots of I-κB kinase/NF-κB signaling showing a negative correlation with GCJ-490A treatment in the normalized enrichment score. The protein levels of IKKα, RelA/p65 and phospho-RelA/p65 (B), the mRNA level of IKKα (C), and the distribution of RelA/p65 (D) in NSCLC cells treated with GCJ-490A. Protein levels of c-Met, RelA/p65, and phospho-RelA/p65 in IKKα knockdown (E) and overexpressing cells (F) were detected by immunoblotting analysis. The relative mRNA levels of *c-Met* in IKKα knockdown (H) and overexpressing cells (G). (I) Western blot analysis showed that knockdown of IKKα reversed the GCJ-490A-mediated decrease in c-Met expression in A549 and HCC827/GR6 cells. All data are representative of at least 3 independent experiments and are presented as mean *±* SD. *P < 0.05; **P < 0.01, 490A: GCJ-490A, p65: RelA/p65, p-p65: phospho-RelA/p65, NC: negative control, mock: plasmid vector control; #1, #2, #3 are 3 different RNAi oligonucleotides.

Although the down-regulation of c-Met induced by HDAC inhibitors has been reported in several studies, the detailed mechanism is far from clear. One published paper has proposed c-Met as a transcriptional target of NF-κB^17^. We performed pathway enrichment analysis of RNA-seq profiling based on GO analysis and GSEA, which also revealed that the IKKα/NF-κB pathway was significantly altered (**Figure 3A**). GSEA indicated that a gene set including *RelA/p65* and 29 other genes (**Supplementary Table S3**) in the IκB kinase/NF-κB signaling pathway were negatively correlated with GCJ-490A treatment in A549 and HCC827/GR6 cell lines (**Figure 3A**). Hence, we assessed alterations in this pathway to identify the mechanism underlying the GCJ-490A-induced down-regulation of c-Met. In agreement with RNA-seq results, a notable up-regulation of IKKα protein was dose dependently induced by GCJ-490A compared with the control in these tested cells, whereas the expression of phosphorylated RelA/p65 and total RelA/p65 was clearly down-regulated by GCJ-490A (**Figure 3B**). Quantitative RT-PCR indicated that the HDAC inhibitor GCJ-490A dramatically elevated IKKα mRNA in these tested cells (**Figure 3C**). Moreover, the protein level of IKKα was markedly increased in xenografts treated with GCJ-490A (**Supplementary Figure S1F**). In addition, nuclear-cytoplasmic isolation assays showed that GCJ-490A down-regulated the expression of RelA/p65 in both the cytoplasm and nucleus (**Figure 3D**). To confirm whether IKKα determines the GCJ-490A-mediated c-Met decline, we transfected cells with siRNA to specifically decrease IKKα levels. As expected, the loss of IKKα dramatically increased c-Met and RelA/p65 expression, particularly the phosphorylation levels of RelA/p65 in the 4 NSCLC cell lines (**Figure 3E**). Furthermore, overexpression of IKKα in A549 and HCC827/GR6 cells led to a decrease in cellular levels of c-Met, RelA/p65, and phosphorylated RelA/p65 (**Figure 3F**). At the transcriptional level, we also found a negative correlation between *IKK*α and c-*Met* in our experimental system (**Figure 3G, 3H**). If the stimulation of IKKα by GCJ-490A is important for c-Met down-regulation, a loss of IKKα would be expected to reverse the GCJ-490A-induced phenotype. We thus suppressed IKKα by shIKKα in A549 and HCC827/GR6 cells, and treated them with GCJ-490A again. As expected, Western blot analysis confirmed that GCJ-490A did not decrease c-Met expression in IKKα-silenced A549 and HCC827/GR6 cells *in vitro* (**Figure 3I**).

### c-Met is repressed by GCJ-490A as a direct target gene of NF-κB

**Figure 4:**
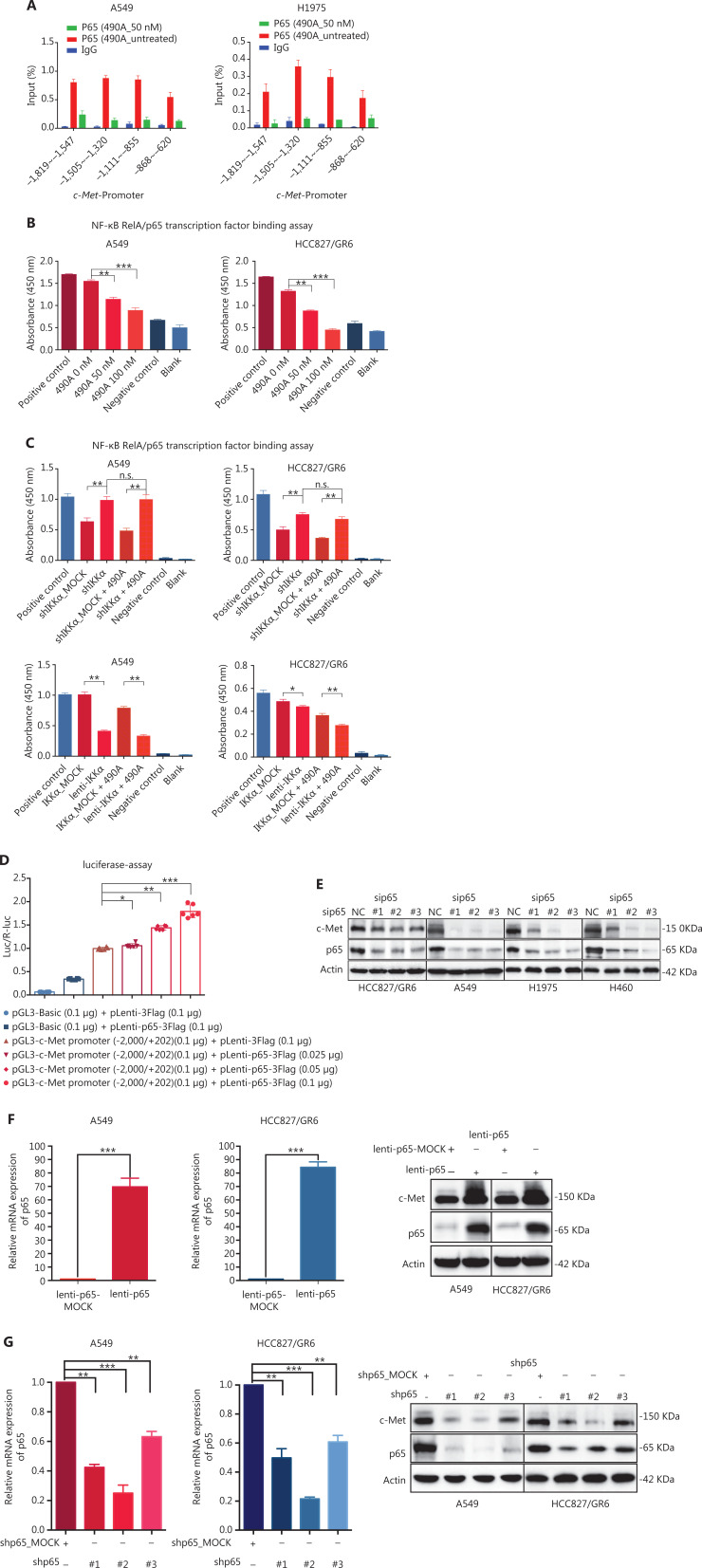
c-Met is repressed by GCJ-490A as a direct target gene of NF-κB. (A) Binding of RelA/p65 to the c-Met promoter was determined by ChIP in cells with or without GCJ-490A treatment. qPCR data are presented as percentage input on the horizontal axis, and the *c-Met* promoter cDNA fragment is shown on the vertical axis. (B) (C) The influence of GCJ-490A on NF-κB RelA/p65 transcription factor binding activity was detected in NSCLC cells and IKKα depleted NSCLC cells. (D) Relative luciferase activities were measured. *c-Met* promoter and *RelA/p65* plasmid were transfected into HEK293T cells (1 × 10^6^ cells). Relative luciferase activity was measured and expressed as a ratio of firefly luciferase to *Renilla* luciferase. The protein level of c-Met in NSCLC cells transfected with sip65 (E), lenti-p65 (F), or shp65 (G), and the mRNA level of p65 in NSCLC cells transfected with lenti-p65 (F) or shp65 (G). All data are representative of at least 3 independent experiments and are presented as *mean ± SD.* *P < 0.05, **P < 0.01, ***P < 0.001. 490A: GCJ-490A, p65: RelA/p65, NC: negative control; #1, #2, #3 are 3 different RNAi oligonucleotides.

c-Met is a transcriptional target of NF-κB, and it participates in NF-κB mediated cell survival in TNFα treated mice and human hepatocarcinoma cells^17^. However, whether c-Met transcriptional regulation by NF-κB is cell type-specific or occurs only after TNFα treatment remains unclear. On the basis of the above results, we hypothesized that GCJ-490A-mediated down-regulation of c-Met occurs through the NF-κB pathway. To test this possibility, we designed primer sequences specific to the *c-Met* promoter and performed ChIP assays. We observed that RelA/p65 was recruited to the *c-Met* promoter, and GCJ-490A significantly suppressed the recruitment in tested cells (**Figure 4A**). Transcription factor activity assays also showed that GCJ-490A effectively suppress the binding of RelA/p65 to DNA polymers (**Figure 4B**), in line with the decreased RelA/p65 level after GCJ-490A treatment. NF-κB transcription factor activity assays also revealed that depletion of IKKα reversed the suppressive effect of GCJ-490A on NF-κB, whereas loss of IKKα alone stimulated NF-κB activity (**Figure 4C**). In agreement with the published data, luciferase reporter assays revealed that exogenous RelA/p65 expression strengthened *c-Met* transcription in our system (**Figure 4D**). Moreover, knockdown of RelA/p65 subunits robustly decreased the mRNA and protein levels of c-Met, whereas transient overexpression of NF-κB increased c-Met expression (**Figure 4E–G**). These results suggest that constitutive activation of c-Met requires RelA/p65 in gefitinib-resistant NSCLC cells. Together, these data support that up-regulation of IKKα by GCJ-490A decreases RelA/p65 expression and transcription activity, and contributes to decreasing c-Met expression.

### GCJ-490A increases histone acetylation at the IKKα promoter and promotes IKKα transcription

**Figure 5:**
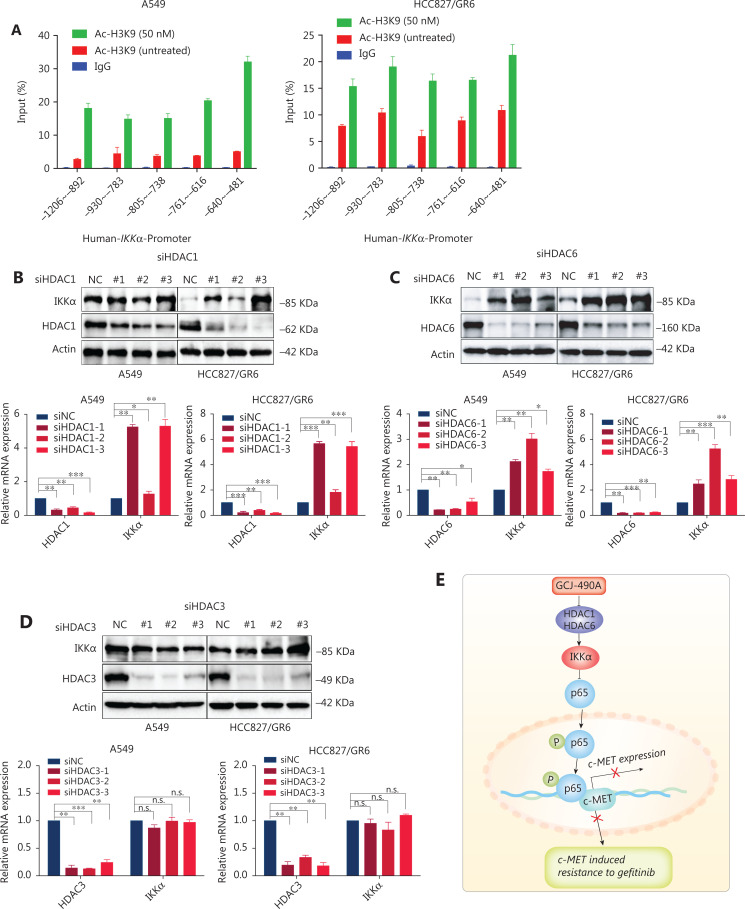
GCJ-490A increases histone acetylation at the *IKK*α promoter and promotes *IKK*α transcription. (A) H3K9 acetylation in the *IKK*α promoter region was observed by ChIP in cells treated with GCJ-490A. (B–D) Western blot for endogenous IKKα in cells with HDAC1, HDAC6, or HDAC3 knockdown. (E) Schematic diagram depicting the proposed mechanism through which GCJ-490A overcomes gefitinib resistance in NSCLC cells. All data are representative of at least 3 independent experiments and are presented as mean ± SD, *P < 0.05, **P < 0.01, ***P < 0.001, p65: RelA/p65, NC: negative control; #1, #2, #3 are 3 different RNAi oligonucleotides.

HDAC inhibitors induce gene expression by up-regulating histone acetylation. To further understand how GCJ-490A regulates IKKα expression, we performed ChIP assays to investigate the HDAC-mediated transcriptional regulation of *IKK*α. As shown in **Figure 5A**, GCJ-490A significantly stimulated H3K9 acetylation recruitment to the region of the *IKK*α promoter in A549 and HCC827/GR6 cells. Given that GCJ-490A, as a pan-HDAC inhibitor, simultaneously inhibits HDAC1, HDAC3, and HDAC6, we next attempted to explore which HDAC family member up-regulates IKKα. A549 and HCC827/GR6 cells were transfected with siRNAs to knock down HDACs (HDAC1, 3, and 6) individually, and HDAC1 and HDAC6 silencing was found to lead to increased IKKα mRNA and protein expression (**Figure 5B–D**).

## Discussion

GCJ-490A is a novel HDAC inhibitor developed by our team, and its anti-tumor efficacy is similar to that of LBH589^18^, a marketed HDAC inhibitor. In our previous studies, we found that GCJ-490A exerts a potent inhibitory effect on colon cancer *in vitro* and *in vivo*^8^. Here, we demonstrated that GCJ-490A displays efficacy in NSCLC treatment. This compound exhibited a broad ability to trigger apoptosis and dramatically repress the proliferation of 8 NSCLC cell lines. The *in vivo* anti-tumor activity of GCJ-490A against NSCLC was moderate, but it did alleviate gefitinib resistance by exerting a remarkable synergistic antitumor effect in combination with gefitinib.

Our RNA-seq profiling data highlight the regulation of the IKK*a*/c-Met pathway by GCJ-490A. Although HDAC inhibitors have been reported to display a synergistic therapeutic effect on EGFR inhibitor resistant NSCLC cell lines when combined with EGFR inhibitors, possibly because of the downregulation of c-Met, the mechanisms remain far from clear. We identified HDAC1 and HDAC6 inhibition as a positive regulator of IKKα expression *via* acetylation of the *IKK*α promoter, which in turn decreases c-Met by suppressing of NF-κB activity. Only one study has reported that c-Met expression may positively correlate with RelA/p65 expression^17^. In addition, c-Met has been reported to accelerate hepatocellular carcinoma tumorigenesis and metastasis *via* activation of NF-κB pathway^19^. We additionally found that c-Met is a direct transcriptional target of RelA/p65. We demonstrated that RelA/p65 is recruited to the *c-Met* promoter region and positively regulates *c-Met* expression. Opinions differ regarding the effects of HDACs on NF-κB activity. Acetylation of RelA/p65, mediated by CBP and p300, is critical for its DNA binding and transactivation activity^20–22^. Some data suggest that deacetylation of RelA by HDAC3 acts as an intranuclear molecular switch that controls the duration of the NF-κB transcriptional response and contributes to replenishment of the depleted cytoplasmic pool of latent-IκBα complexes^23^. In contrast, deacetylation of RelA/p65 by HDACs, including HDAC1, HDAC2, HDAC3, and SIRT1, has been reported to repress its transcriptional activity^24,25^. In agreement with these results, inhibitors of deacetylases, such as TSA and sodium butyrate, have been reported to potentiate TNF-induced NF-κB activation^26^. TSA acetylated RelA/p65 prolongs both the TNF-induced DNA-binding activity and the presence in the nucleus of RelA/p65. However, we found that GCJ-490A significantly repressed nuclear RelA/p65 and its DNA-binding activity *via* upregulation of IKKα expression. Some evidence indicates that IKKα activates NF-κB-dependent gene transcription by enhancing transactivation and DNA binding of RelA/p65^22,27,28^. Ganor’s group has further shown that IKKα functions as a chromatin kinase in the nucleus and targets histone H3 at Ser10, thus activating NF-κB-directed gene expression^22,29^. However, the results across studies are not consistent. IKKα has been described as a negative regulator of NF-κB activity by accelerating the turnover of the NF-κB subunits RelA and c-Rel, and their removal from target gene promoters^30^. In agreement with that study, we demonstrated that IKKα overexpression suppressed NF-κB activity, decreased binding of RelA/p65 to *c-Met* gene promoters, and suppressed *c-Met* transcription in NSCLC cells. In contrast, IKKα depletion activated NF-κB activity and increased *c-Met* transcription. The establishment of the HDACs-IKKα-RelA/p65 axis provides better understanding of molecular pathways through which deacetylases regulate transcriptional activity, which may facilitate the development of more effective cancer therapies.

Despite recent progress in the treatment of NSCLC with the approval of EGFR inhibitors as first-line therapy, resistance to these inhibitors always occurs after a median duration of 9–15 months^31^, thus necessitating the development of more efficient therapeutics. *c-Met* gene amplification and/or protein hyper-activation are known to be key mechanisms of acquired resistance to EGFR inhibitors. The dramatic inhibition of c-Met expression by GCJ-490A, and the favorable distribution of GCJ-490A in the lungs inspired us to explore potential benefits of its use in combination with EGFR inhibitors. A benefit to the use of HDAC inhibitors in combination with EGFR inhibitors (to increase EGFR inhibitor efficacy or decrease tumor resistance) has been observed in clinical trials. The Wisconsin Oncology Network phase II study has shown that monotherapy with vorinostat in patients with relapsed NSCLC provides substantial benefits regarding time to progression^32^. Panobinostat has recently been demonstrated to sensitize EGFR-mutated and wild-type NSCLC cells to the anti-proliferative activity of erlotinib. In addition, a small phase I trial of combination therapy with panobinostat and EGFR inhibitor in patients with advanced NSCLC has shown that this therapeutic regimen is well tolerated. Larger randomized controlled studies are underway to elucidate the benefits in erlotinib-resistant NSCLC^33^. Notably, we observed that 4 gefitinib-resistant NSCLC cell lines, which have different resistance mechanisms (including c-Met amplification in HCC827/GR6, KRAS mutation in A549 or HCC827/GR6, and EGFR T790M/L858R mutation in H1975), were effectively inhibited by the combination treatment with GCJ-490A and gefitinib *in vitro* and *in vivo*. In addition, readily measurable apoptosis was induced by co-treatment at doses well below those of either GCJ-490A or gefitinib alone required to induce apoptosis, thus indicating that these agents have higher potency and lower toxicity when used in combination. Although a potential synergistic therapeutic effect of HDAC inhibition together with EGFR inhibition against EGFR inhibitor-resistant NSCLC cell lines has been reported in many studies, the mechanisms are unclear. Cancer cells may be sensitized to gefitinib by HDAC inhibitors, an effect that may be associated with the up-regulation of Bim^34,35^ and the down-regulation of EGFR and c-Met. In our experiments, combined treatment promoted cell apoptosis. In addition, we elucidated that HDAC inhibition down-regulates c-Met expression in a manner dependent on IKKα up regulation, according to several assays, and this effect may be one of the main mechanisms through which HDAC inhibitors overcome resistance to gefitinib. In this study, we report the first evidence that HDAC inhibition, specifically inhibition of HDAC1 and HDAC6, plays an upstream regulatory role in IKKα expression, thus leading to c-Met downregulation, which subsequently overcomes gefitinib resistance in NSCLC. These results provide a clear rationale for pursuing the clinical evaluation of GCJ-490A and other HDAC inhibitors in combination with EGFR inhibitors in NSCLC treatment.

## Conclusions

In summary, we identified that the novel pan-HDAC inhibitor GCJ-490A exerts potent anti-tumor activity against NSCLC. More importantly, we demonstrated an IKKα/c-Met signaling pathway that is induced by HDAC inhibitors and has potential for overcoming gefitinib resistance. Our findings suggest clinical benefits of combining HDAC inhibitors and EGFR inhibitors in NSCLC treatment. These data provide a rational basis for testing GCJ-490A in combination with EGFR inhibitors in clinical trials.
