# Supplementary material for: The HDAC inhibitor GCJ-490A suppresses c-Met expression through IKKα and overcomes gefitinib resistance in non-small cell lung cancer
Source: Cancer Biol Med. 2022 Feb 22;19(8):1172–92. doi: 10.20892/j.issn.2095-3941.2021.0130 (PMC9425179; doi:10.20892/j.issn.2095-3941.2021.0130)
Supplement: Supplementary file 1 [file cbm-19-1172-s001.pdf]

Supplementary materials

Table S1 The most significantly downregulated genes in A549 in the heatmap

Gene name	Gene name
PIGB	CDH2
ITGAE	PARPBP
SEPHS1	SMARCB1
HSD17B14	E2F3
VAV3	NT5C
ZFHX3	MPST
RNPEPL1	NEK3
LARP1	PPRC1
LRP5	STK32B
ERN1	PWWP2B
ZNRF3	ODF3B
MBP	DNAJC17
TNFRSF10A-AS1	EDN2
PDE8A	RNF217
XIAP	LPAR5
GABRE	RTN4RL1
CHRAC1	AC011604.2
MET	C17orf113
TBL2	AL590399.1
SLC22A18	AC004585.1
NEK2	AC090984.1
EIF2S2	AC013717.1
CANX	LYSMD2
SGCE	PAK1
FAM98C	MIA2
TXLNGY	PRKCA

Table S2 The most significantly downregulated genes in HCC827/GR6 in the heatmap

Gene name	Gene name
IRAK1	AL031587.5
GPX1	KCNMB2-AS1
MSI2	NQO1
SUMO3	KLF8
MET	HOXB3
INPPL1	AL683813.1
PTPRF	KCNIP3
FAM219B	CNTNAP3C
ARID1B	WHAMMP2
CRTC3	SUCLG2-AS1
CHEK1	CDK15
EPDR1	BX322639.1
DBF4	ACOT1
ACCS	REC8
PLPP2	ZNF761
PDGFA	PCDHB12
IFI35	TUBB2B
SLC18B1	TMSB15B
BANK1	ITPKA
ADCY6	AL078644.2
TMEM131	MAP3K15
H6PD	MRPS30-DT
SNX33	ITGB3
MGLL	AP000233.1
NOTCH1	CTSO
KIAA0040	VIPR1
TLR6	GDPD3
SLC9A5	

Table S3 GSEA analysis details

Gene ID	Gene name	Rank metric score	Running ES	Core enrichment
ENSG00000135341	MAP3K7	−0.52269	−0.3752357	Yes
ENSG00000136560	TANK	−0.54068	−0.3723594	Yes
ENSG00000170315	UBB	−0.60349	−0.3750947	Yes
ENSG00000154589	LY96	−0.63725	−0.3732641	Yes
ENSG00000175104	TRAF6	−0.66051	−0.3689179	Yes
ENSG00000126895	AVPR2	−0.68421	−0.3640947	Yes
ENSG00000142867	BCL10	−0.68609	−0.3562919	Yes
ENSG00000221995	TIAF1	−0.71607	−0.3520593	Yes
ENSG00000102871	TRADD	−0.74947	−0.3478177	Yes
ENSG00000157625	TAB3	−0.77264	−0.34162647	Yes
ENSG00000173366	AC097637.1	−0.82206	−0.33789715	Yes
ENSG00000092098	RNF31	−0.96714	−0.34297	Yes
ENSG00000173039	RELA	−1.30400	−0.35660937	Yes
ENSG00000104856	RELB	−1.43169	−0.34912977	Yes
ENSG00000197885	NKIRAS1	−1.48264	−0.3352379	Yes
ENSG00000269335	IKBKG	−1.50343	−0.31922486	Yes
ENSG00000010671	BTK	−1.57812	−0.30587628	Yes
ENSG00000100906	NFKBIA	−1.69370	−0.29363322	Yes
ENSG00000127191	TRAF2	−1.74969	−0.27596563	Yes
ENSG00000232810	TNF	−1.98256	−0.26460975	Yes
ENSG00000162924	REL	−2.05991	−0.2441729	Yes
ENSG00000196664	TLR7	−2.14359	−0.22242361	Yes
ENSG00000137275	RIPK1	−2.14598	−0.19735643	Yes
ENSG00000187796	CARD9	−2.32041	−0.17764041	Yes
ENSG00000239732	TLR9	−2.46754	−0.15404114	Yes
ENSG00000006062	MAP3K14	−2.80945	−0.13223644	Yes
ENSG00000170458	CD14	−2.81081	−0.09936612	Yes
ENSG00000077150	NFKB2	−3.17617	−0.07228604	Yes
ENSG00000163877	SNIP1	−4.24796	−0.04370275	Yes
ENSG00000023445	BIRC3	−4.75435	0.003838288	Yes

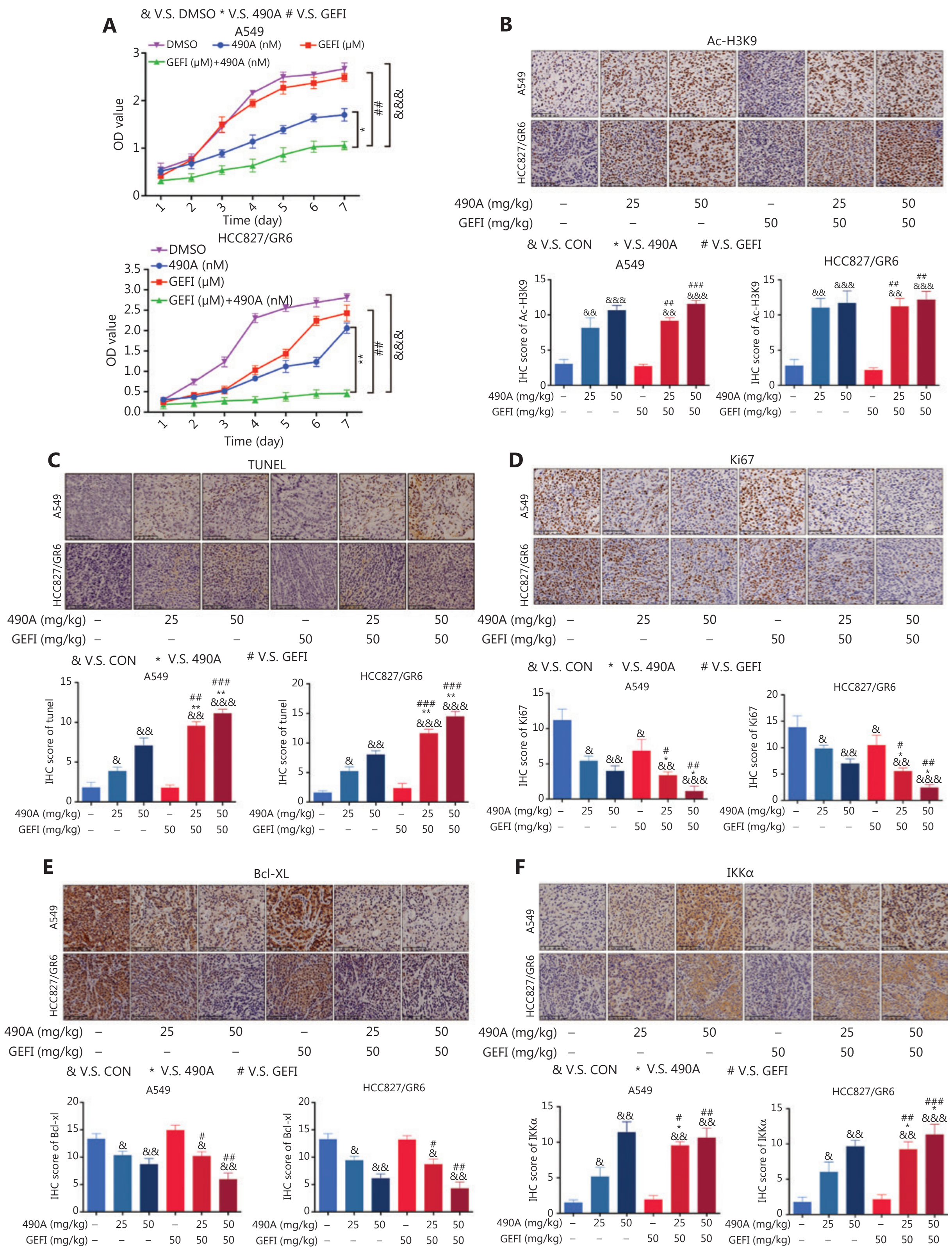
A
& V.S. DMSO * V.S. 490A # V.S. GEFI
A549
DMSO
490A (nM)
GEFI (μM)
GEFI (μM)+490A (nM)
OD value
Time (day)
HCC827/GR6
B
Ac-H3K9
A549
HCC827/GR6
490A (mg/kg) – 25 50 – 25 50
GEFI (mg/kg) – – – 50 50 50
& V.S. CON * V.S. 490A # V.S. GEFI
IHC score of Ac-H3K9
C
TUNEL
IHC score of tunel
D
Ki67
IHC score of Ki67
E
Bcl-XL
IHC score of Bcl-xl
F
IKKα
IHC score of IKKα

Figure S1 (A) Growth curves of HCC827/GR6 and A549 cells treated with the indicated compounds. The levels of acetyl-H3K9 (B), TUNEL (C), Ki67 (D), Bcl-XL (E), and IKKα (F) in tumor tissues were detected with IHC (scale bar 100 μm). All data were analyzed with 2-tailed Student's *t*-test and are presented as mean ± SD, $^{*}P < 0.05$; $^{**}P < 0.01$; $^{\#}P < 0.05$; $^{\#\#}P < 0.01$; $^{\#\#\#}P < 0.001$; $^{\&}P < 0.05$; $^{\&\&}P < 0.01$; $^{\&\&\&}P < 0.001$, 490A: GCJ-490A, GEFI: gefitinib.

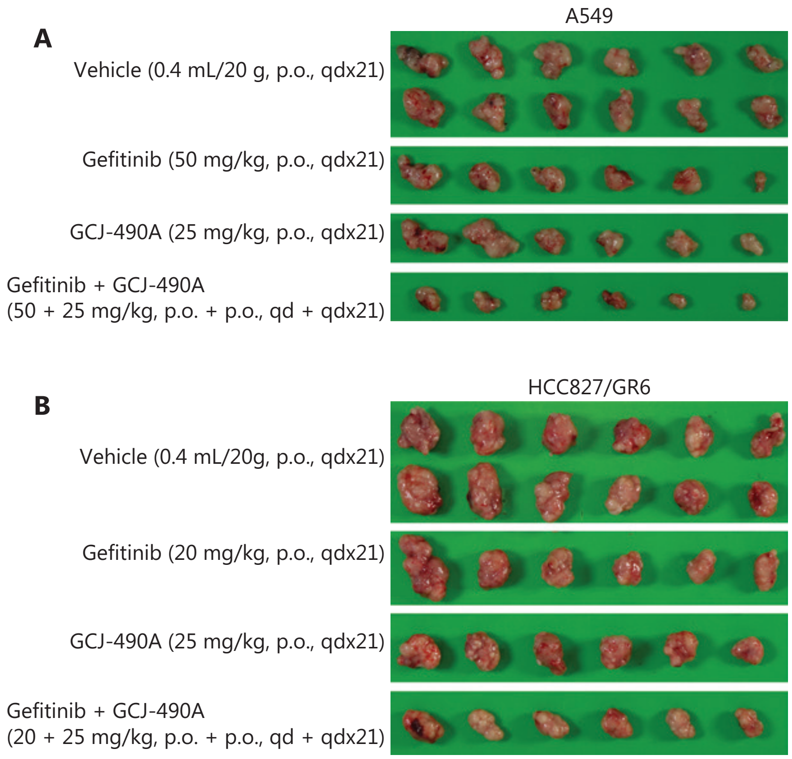

Figure S2 Representative images of tumors from A549 (A) and HCC827/GR6 (B) xenograft mice in the vehicle and treated groups are displayed.
